# Characteristics and potential malignancy of colorectal juvenile polyps in adults: a single-center retrospective study in China

**DOI:** 10.1186/s12876-022-02151-x

**Published:** 2022-02-21

**Authors:** Jie Dong, Tian-Shi Ma, Yuan-Hong Xu, Peng Li, Wan-Yuan Chen, Jiang-Feng Tu, You-Wei Chen

**Affiliations:** 1 Cancer Center, Department of Gastroenterology, Zhejiang Provincial People’s Hospital (Affiliated People’s Hospital, Hangzhou Medical College), No. 158, Shangtang Road, Hangzhou, 310014 Zhejiang Province China; 2 Cancer Center, Department of Pathology, Zhejiang Provincial People’s Hospital (Affiliated People’s Hospital, Hangzhou Medical College), No.158, Shangtang Road, Hangzhou, 310014 Zhejiang Province China; 3 Department of Gastroenterology, The Fifth Affiliated Hospital of Zunyi Medical University, Zhuhai, 519000 Guangdong Province China

**Keywords:** Colorectal juvenile polyps in adult patients, Characteristics, Potential malignancy, Chicken skin mucosa, Treatment

## Abstract

**Background:**

Colorectal juvenile polyps are rare and generally considered benign in adults. Carcinogenesis or neoplastic changes are rarely mentioned in the literature. We systematically evaluated the characteristics and potential malignancy of colorectal juvenile polyps in adults.

**Methods:**

We retrospectively reviewed the medical records of 103 adults diagnosed with colorectal juvenile polyps from September 2007 to May 2020 at our hospital. The characteristics, endoscopic findings, occurrence of intraepithelial neoplasia, carcinogenesis and diagnostic value of chicken skin mucosa (CSM) were analyzed.

**Results:**

The average age of patients with juvenile polyps was 43.2 years (range, 19 to 78 years). A total of 101 patients (101/103, 98.1%) had a single juvenile polyp, and two patients had multiple polyps (107 polyps in total). Polyp sizes ranged from 0.5 to 5 cm. One (1/107, 0.9%) juvenile polyp was cancerous, and 7 (7/107, 6.5%) developed low-grade intraepithelial neoplasia. Neoplasia and cancerization did not appear in the two patients with multiple polyps. A 27-year-old female had a 2-cm polyp with well-differentiated adenocarcinoma in the mucosa in the sigmoid colon with erosion on the surface. CSM was observed adjacent to 17 polyps, which were all located in the rectum and sigmoid colon, and one polyp showed low-grade intraepithelial neoplasia.

**Conclusions:**

Colorectal juvenile polyps occur in a wide range of locations and in variable sizes and numbers. These polyps are solitary in most patients and have neoplastic potential. CSM is not a tumorigenic marker in colorectal juvenile polyps and usually occurs in the distant colorectum. Colorectal juvenile polyps in adults may progress from low-grade intraepithelial neoplasia to high-grade intraepithelial neoplasia and then to carcinoma and should be treated when discovered and regularly followed as colorectal adenomas.

## Background

Juvenile polyps are a type of hamartoma. Although these polyps are the most common type in children [1], colorectal juvenile polyps are rare in adults [2, 3]. The occurrence rate of juvenile polyps in children and adolescents is 2%, which accounts for the majority (approximately 80–90%) of polyps in paediatric patients [1, 3, 4]. Hyperplastic polyps and adenomas are the two most common types of polyps in adults. Less than 1% of juvenile polyps occur in adults [5], and there have been few studies on juvenile polyps in adults. Juvenile polyposis syndrome (JPS) is generally characterized by multiple hamartomatous polyps throughout the gastrointestinal tract, and it is considered an autosomal dominant disorder. JPS is accompanied by an increased risk of colorectal and gastric cancer [6, 7]. Unlike JPS, sporadic juvenile polyps in the colon are often solitary and rarely undergo malignant transformation [1, 4, 8]. However, sporadic juvenile polyps may also exhibit dysplasia [5, 9, 10]. Previous studies have primarily focused on juvenile polyps in children. However, juvenile polyps in adults are rare and have been less investigated. Except for research from Denmark [5], there have been few studies of adult colorectal juvenile polyps in large populations, especially in the Asia–Pacific area. We performed a retrospective study of adult patients diagnosed with colorectal juvenile polyps in a Chinese population at a single center. Demographic characteristics, clinical symptoms, endoscopic manifestations and pathological results were analyzed. This study summarized the characteristics and evaluated the potential malignancy and carcinogenic factors of colorectal juvenile polyps in adults.

## Methods

### Study design

A retrospective analysis of the clinical and pathological data of adult patients diagnosed with colorectal juvenile polyps who were admitted to our clinic from September 2007 until May 2020 was performed. The following inclusion criteria were used: (1) age older than 18 years at the time of diagnosis; and (2) pathological diagnosis of colorectal juvenile polyps. The exclusion criteria were juvenile polyposis syndrome (JPS), Cronkhite–Canada syndrome (CCS) or other types of polyposis. Patient age at initial diagnosis (years), sex, abdominal pain, diarrhoea, haematochezia, mucus in the stool and other clinical manifestations, the number, maximum diameter (cm), position, polymorphic morphology of polyps defined by the Paris classification [11] [pedunculated type (0-Ip), subpedunculated type (0-Isp) and sessile type (0-Is)], endoscopic features such as mucosal changes near polyps and pathological results were collected and analyzed. Chi-square tests were used to compare detection rates between groups. Spearman rank correlation analyses were used to compare the characteristics of polyps and clinical features. The expression of MutL homologue 1 (MLH1), MutS homologue 2 (MSH2), MutS homologue 6 (MSH6) and postmeiotic segregation increased 2 (PMS2) in the samples was tested using immunohistochemical staining with the EnVision two-step procedure. Ethics approval and consent to participate for the study was obtained from the Ethics Committee of Zhejiang Provincial People's Hospital (IRB No. 2020QT239).

## Results

### Subjects

From 9/2007 to 5/2020, a total of 103 patients with 107 juvenile polyps were included in this study. Patients were divided into two groups according to the number of polyps (1 polyp and 2–4 polyps). A total of 101 patients (98.1%) had a single juvenile polyp, and two patients had multiple polyps. Sixty-four patients were male, and 39 were female. The median age was 43.2 years (range 19–78 years). None of the patients had a family history of polyposis. Three patients had a history of cancer. Two of these patients had colon cancer, and one patient had appendix cancer long before they were diagnosed with colorectal juvenile polyps. A history of cancer had no clinical relevance to the incidence of juvenile polyps. One hundred patients were treated with endoscopy. A total of 35.8% (19/53) of patients had positive faecal occult blood test results (Table 1).

**Table 1:** Summary of patients’ conditions and medical procedures

Characteristic	
Age, years	
Mean	43.2
Range	19–78
Sex, n	
Male	64
Female	39
Clinical manifestations, n	
Abdominal pain	18
Diarrhoea	11
Bloody stool	45
Mucous stool	5
Numbers of polyps, n	
Single	101
Multiple	2
Location, n	
Ileocecum	1
Ascending colon	10
Transverse colon	10
Descending colon	13
Sigmoid	38
Rectum	35
Gross appearance, n	
Paris 0-Is	18
Paris 0-Isp	39
Paris 0-Ip	50
Maximum diameter (cm), n	
0–0.9	34
1–1.9	43
2–2.9	19
≥ 3	11
Chicken skin mucosa, n	
With	17
Without	90
Pathological results, n	
With low-grade intraepithelial neoplasia	7
With cancerization	1
Therapy, n	
Endoscopic therapy	100
Surgery	3

### Clinical manifestations

Among the 103 patients, 18 (16.8%) patients complained of abdominal pain, 11 (10.3%) patients visited doctors for diarrhoea, 45 (42.1%) patients experienced bloody stool, and 5 (4.7%) patients had mucus in their stool. Thirty-five (32.7%) patients had no complaints. Juvenile polyps were found on colonoscopy examination (Table 1).

### Endoscopic features

A total of 101 patients (98.1%) had a solitary juvenile polyp, and two patients had three polyps. Of the 107 polyps found, most were located in the sigmoid (38, 35.5%) and rectum (35, 32.7%), while 1 (0.9%) polyp was in the ileocecum, 10 (9.3%) polyps were in the ascending colon, 10 (9.3%) polyps were in the transverse colon, and 13 (12.1%) polyps were in the descending colon. The size of the polyps ranged from 0.5 to 5.0 cm. The majority (43; 40.2%) of polyps were 1–1.9 cm, but 34 (31.8%) polyps were less than 1 cm, 19 (17.8%) polyps were from 2.0 to 2.9 cm, and 11 (10.3%) polyps were larger than 3 cm. Eighteen (16.8%) polyps were Paris 0-Is polyps, 39 (36.4%) polyps were Paris 0-Isp polyps, and the other 50 (46.7%) were Paris 0-Ip, which accounted for the majority of polyps. Fifty-four (50.5%) polyps showed a reddish surface. Chicken skin mucosa (CSM) was observed adjacent to 17 polyps, which were all located in the rectum and sigmoid colon and accounted for 23.2% (17/73) of all rectosigmoid juvenile polyps (Table 1). Patient age, sex, abdominal pain, and diarrhoea were not associated with polyp location, size or polymorphic morphology. Haematochezia was positively correlated with polyp size (*P* < 0.001) and polymorphic morphology (*P* = 0.005). Mucus in the stool was positively correlated with polyp size (*P* = 0.001). 0-Ip polyps were more likely to be associated with haematochezia than 0-Isp and 0-Is polyps (Table 2).

**Table 2:** Spearman rank correlation analyses of characteristics of polyps and clinical features

	Location	Size	Paris classification
Age	*P* = 0.056	*P* = 0.866	*P* = 0.577
Sex	*P* = 0.883	*P* = 0.061	*P* = 0.504
Abdominal pain	*P* = 0.240	*P* = 0.674	*P* = 0.617
Diarrhoea	*P* = 0.647	*P* = 0.431	*P* = 0.992
Haematochezia	*P* = 0.828	*P* < 0.001	*P* = 0.005
Mucus in the stool	*P* = 0.436	*P* = 0.001	*P* = 0.774

### Treatment

One hundred patients underwent endoscopic therapy, including thermal biopsies, loop snare techniques or endoscopic mucosal resection (EMR), and three patients underwent surgery. One patient was diagnosed with colon cancer before surgery based on the morphology of the mass on endoscopy, but the postoperative specimen was identified as a juvenile polyp with high levels of stromal oedema and focal tubular adenoma with low-grade intraepithelial neoplasia. The polyp was located in the descending colon and had a maximum diameter of 5.0 cm. One patient complaining of abdominal pain and diarrhoea was diagnosed with colon cancer before surgery and underwent radical surgery. The third patient had three large polyps approximately 5.0 cm in diameter that could not be treated under endoscopy (Table 1).

### Pathological results

**Fig. 1:**
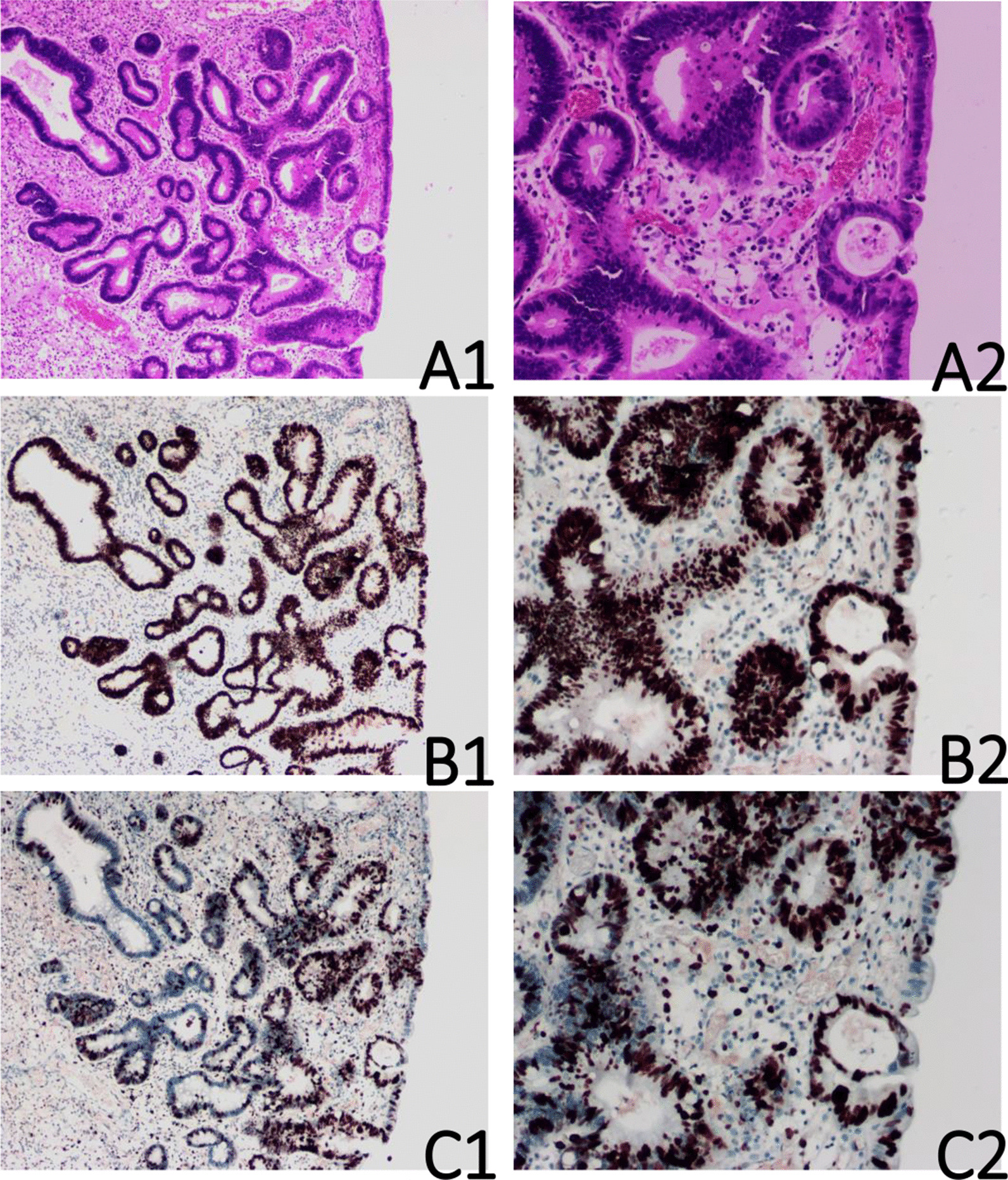
Juvenile polyp with carcinogenesis. **A** Haematoxylin and eosin staining (A1 × 40; A2 × 100). **B** Immunohistochemical staining for p53 showed p53 mutation and overexpression (B1 × 40; B2 × 100). **C** Immunohistochemical staining for Ki-67 showed an index of 80% (C1 × 40; C2 × 100)

**Fig. 2:**
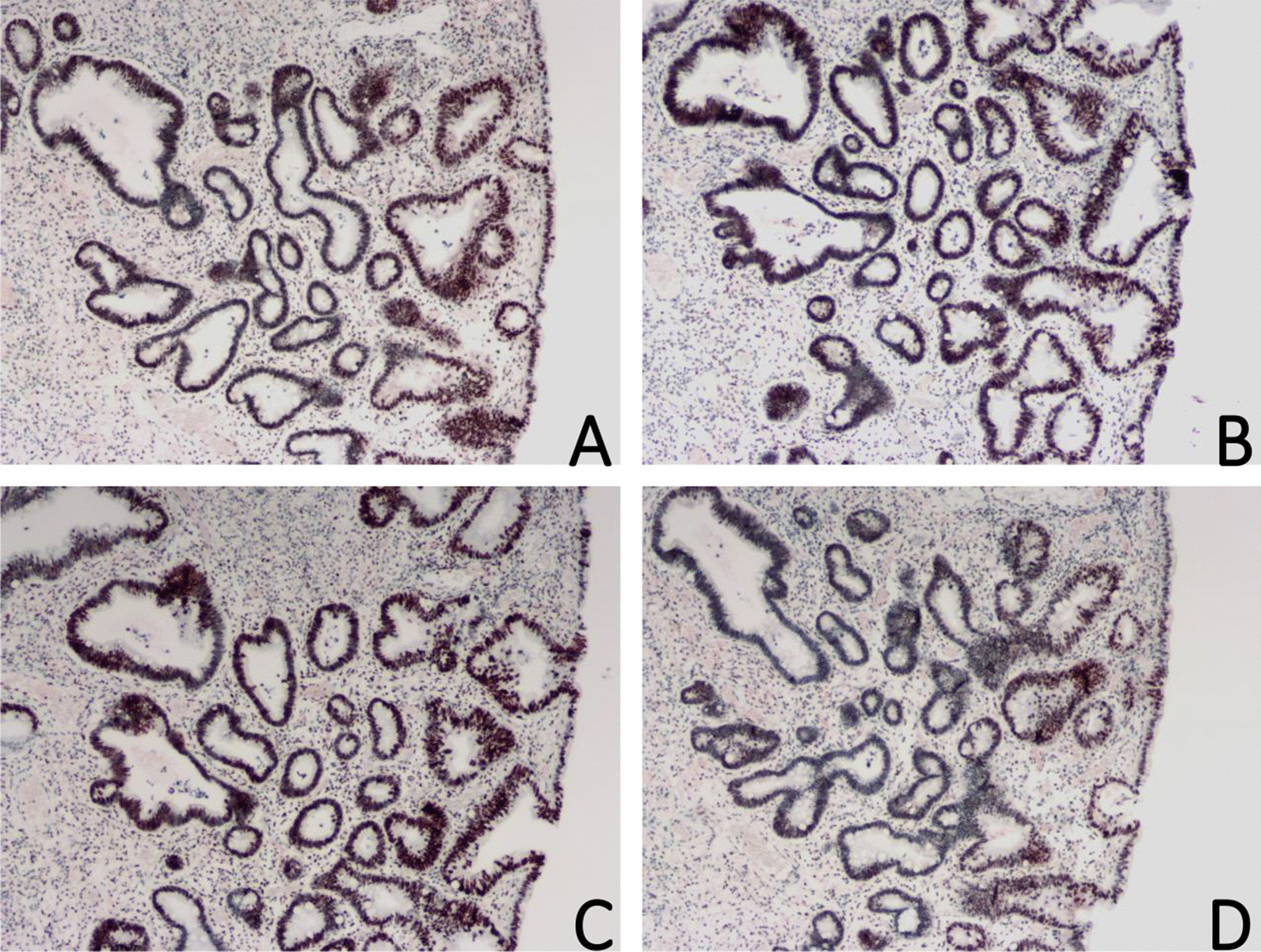
Juvenile polyp with carcinogenesis. Immunohistochemical staining was positive for **A** MLH1 (× 40), **B** MSH2 (× 40), **C** MSH6 (× 40), and **D** PMS2 (× 40)

**Fig. 3:**
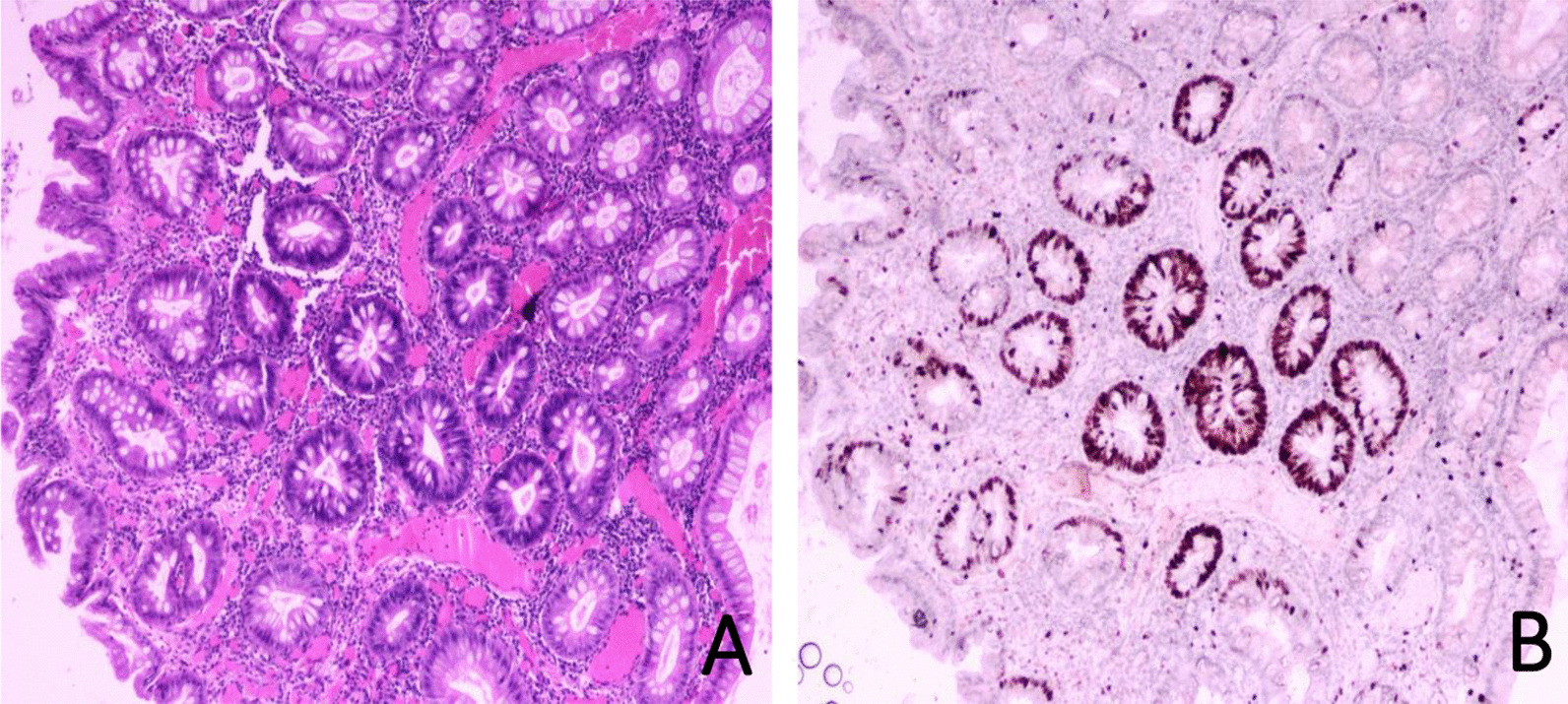
Juvenile polyp with low-grade intraepithelial neoplasia. **A** Haematoxylin and eosin staining (× 40). **B** Immunohistochemical staining for Ki-67 showed an index of 40% (× 40)

**Fig. 4:**
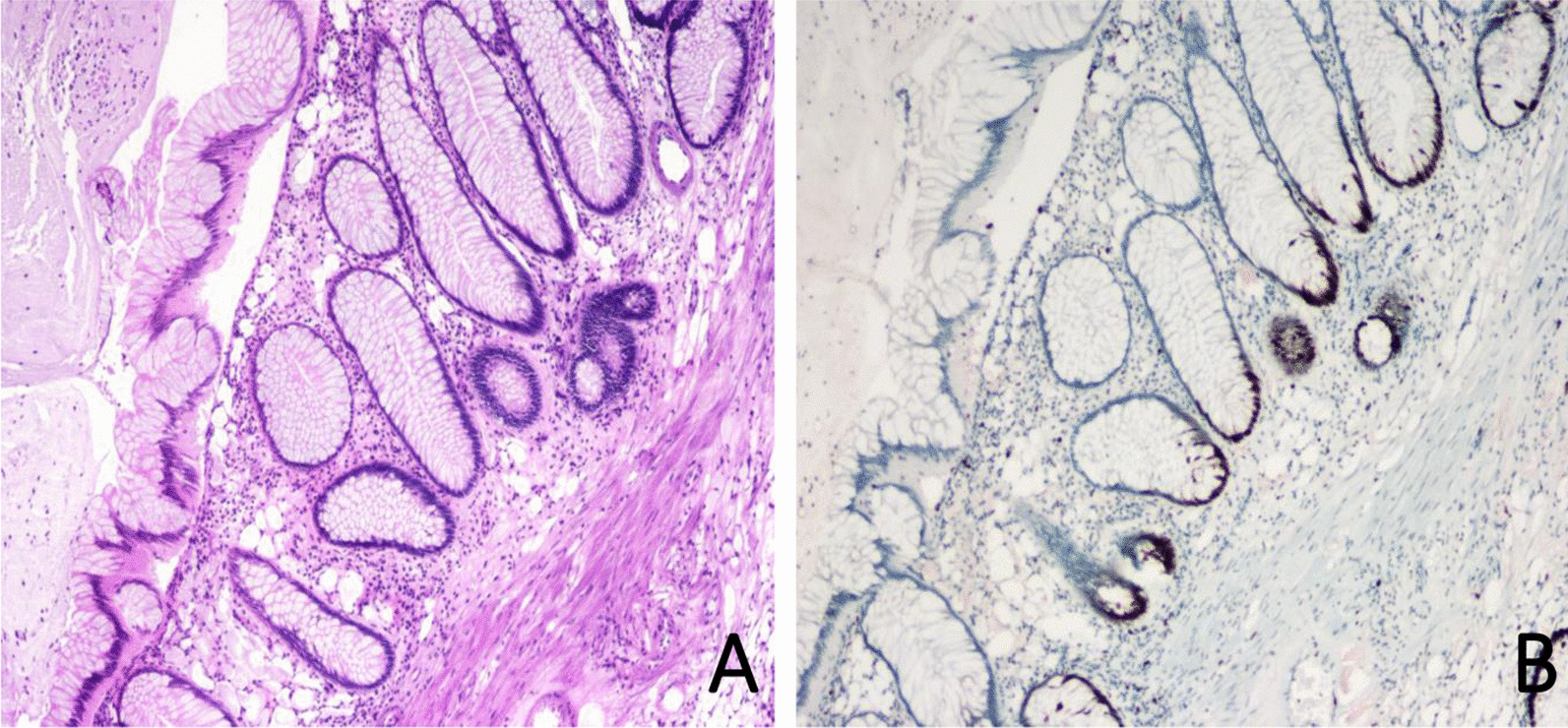
Simple juvenile polyp. **A** Haematoxylin and eosin staining (× 40). **B** Immunohistochemical staining for Ki-67 showed an index of 20% (× 40)

One colorectal juvenile polyp showed focal carcinogenesis, and seven polyps showed low-grade intraepithelial neoplasia. These polyps were all single polyps. Multiple juvenile polyps were not accompanied by cancerization. There were two tubular adenomas with low-grade intraepithelial neoplasia near two juvenile polyps. One adenoma was accompanied by colon cancer. The patient with focal carcinogenesis was a 27-year-old female. Her polyp, which was approximately 2.0 cm in size, was located in the sigmoid colon and showed erosion on the surface. Immunohistochemical staining of the polyp showed a Ki-67 index of approximately 80%, mutated p53, with diffuse and strongly positive expression, and positive expression of MLH1, MSH2, MSH6 and PMS2, indicating microsatellite stability (Figs. 1, 2). The immunohistochemical results of the seven polyps with low-grade intraepithelial neoplasia showed an average Ki-67 index of approximately 40% (Fig. 3), wild-type p53 without overexpression, and MLH1, MSH2, MSH6 and PMS2 expression. The Ki-67 index was approximately 20% (Fig. 4) in other simple juvenile polyps, with wild-type p53 and no overexpression. Among the 17 polyps with CSM, one (5.9%, 1/17) polyp showed low-grade intraepithelial neoplasia. Six polyps with low-grade intraepithelial neoplasia and one polyp with carcinogenesis were found among the remaining 90 polyps without CSM. The chi-square test did not show a significant difference (P > 0.05) (Table 1).

## Discussion

To the best of our knowledge, the current study is the largest single center study on colorectal juvenile polyps in adults in the Asia–Pacific area. Colorectal juvenile polyps are rare in adults. The incidence of juvenile polyps in Danish adults ranges from 1:65,000 to 1:40,000 [5]. Based on our study, the average age of onset in adults was 43.2 years. The ratio of males to females was 1.6:1. These findings are consistent with those of previous reports, indicating an average age between 25.5 and 48.9 years [5, 12] and a male:female ratio of 0.8–1.4:1 [8].

The clinical manifestations of juvenile polyps are similar to those of other types of polyps and include abdominal pain, rectal bleeding, prolapse, and diarrhoea. Juvenile polyps can occur in any part of the colon, but most polyps in the current study were located in the rectum and sigmoid colon (68.2%), which is similar to the distribution of juvenile polyps reported in children [13] and in a previous study in adults [5]. A total of 98.1% (101/103) of the patients had a single juvenile polyp, which is similar to the rate of 94.9% reported previously [5]. A total of 42–75.9% of children had a single polyp [13, 14]. Additionally, 83.2% of the polyps were Paris 0-Isp/Ip according to the Paris endoscopic classification [15, 16] in our study, which is similar to the finding of a previous report [17].

The size of juvenile polyps ranged from sessile nodules of a few millimetres to pedunculated lesions up to several centimetres, as determined by endoscopy. Large polyps may be multilobulated, but small polyps are generally round and smooth. Erosion and granulation tissue hyperplasia are often observed on the surface of polyps [18]. Haematochezia and mucus in the stool were correlated with a larger juvenile polyp size, and 0-Ip polyps were more likely to be associated with haematochezia than 0-Isp and 0-Is polyps by the Paris classification. Small yellow particles were also observed around some juvenile polyps; this manifestation is referred to as CSM [19].

Histopathology is the gold standard for the diagnosis of juvenile polyps because the clinical symptoms and endoscopic features are not entirely typical. This condition primarily manifests as mucinous gland hyperplasia and mucous cysts of different sizes in fibrous tissues. Juvenile polyps are composed of differentiated glandular ducts, and the glandular cavity is dilated to varying degrees. This dilation is generally accompanied by interstitial hyperplasia and the infiltration of large numbers of inflammatory cells, such as lymphocytes, plasma cells, neutrophils and eosinophils, in the stroma. These characteristics distinguish juvenile polyps from juvenile polyposis and Peutz–Jeghers syndrome.

Juvenile polyps are a type of hamartoma with minimal risk [8, 20, 21]. However, the potential of solitary or sporadic juvenile polyps to develop into cancer is not clear. Only a few cases of carcinogenesis from solitary or sporadic juvenile polyps have been described in the literature. Intramucosal carcinoma arising within a solitary juvenile polyp is regarded as ‘a wolf in sheep’s clothing’ [17, 22]. Other researchers [10, 23, 24] reported three cases of signet ring cell carcinoma in juvenile polyps. One (0.9%) juvenile polyp with focal carcinogenesis and seven (6.5%) polyps with low-grade intraepithelial neoplasia were identified in our study. These polyps were all single polyps. Neoplasia or cancerization did not appear in the two patients with multiple polyps, which is consistent with the finding in a previous report in children [14] that increased numbers of polyps at presentation did not predict further polyp development.

The incidence of adenomatous changes in juvenile polyps is not clear. The polyp with focal carcinogenesis showed higher Ki-67 and p53 expression levels than the seven polyps with low-grade intraepithelial neoplasia. These seven polyps showed higher Ki-67 expression than simple polyps. As previously reported, the expression of p53 and Ki-67 may be used as prognostic factors for adenomas, with high cell proliferation suggesting more aggressive behaviour. Higher levels of p53 and Ki-67 expression are found in adenomas with high-grade dysplasia [25, 26].

Based on the results of the immunohistochemical markers mentioned above, we hypothesized that juvenile polyps progress from low-grade intraepithelial neoplasia to high-grade intraepithelial neoplasia and then to carcinoma. A study of 213 paediatric patients found adenomatous changes that were suggestive of the same progression [14]. Based on these findings, the risk of carcinogenesis and the route of cancerization are independent of age and the number of polyps. Based on previous studies and our research, sporadic juvenile polyps might carry an inherent potential for malignancy.

CSM was first identified in 1998 [27] and was described as specific mucosal morphological changes adjacent to colorectal neoplasms. CSM is characterized by a speckled pattern of pale-yellow colorectal mucosa on endoscopy. The prevalence of CSM was reported to be 30.7% (225/733) in patients with adenomas. Adenomas with CSM exhibited more high-grade dysplasia and carcinoma than adenomas without CSM, higher expression of Ki-67, COX2 protein and survivin, and lower expression of caspase-3, which indicated the carcinogenetic progression of colorectal adenomas [28, 29]. Therefore, CSM is generally considered a tumour marker in colorectal adenomas. In contrast, since the level of Ki-67 or p53 expression are not increased in juvenile polyps with CSM in children, CSM is not regarded a marker for subsequent malignancy [30, 31]. In our study, there was no difference in the incidence of neoplasia or tumorigenesis between polyps with or without CSM. Hence, CSM was not identified as a tumorigenic marker of colorectal juvenile polyps as it has been in children.

Endoscopic polypectomy is the main treatment for colorectal juvenile polyps. Thermal biopsies, loop snare techniques, EMR, and endoscopic submucosal dissection (ESD) are safe and effective for sporadic, semipedunculated or sessile large juvenile polyps. However, colectomy may be beneficial for multiple or diffuse juvenile polyps, very large polyps, or polyps suspected of malignant transformation [32, 33]. Even though juvenile polyps in adults are rare, they should be treated when discovered, with regular follow-up as for colorectal adenomas, as improved compliance with follow-up reduces the risk of carcinogenesis.

## Conclusion

This study is the largest single-center study of the characteristics and potential malignancy of colorectal juvenile polyps in adults in the Asia–Pacific area. Even though colorectal juvenile polyps are often considered benign, they still carry a risk of malignancy. We found a 0.9% (1/107) incidence of cancer and a 6.5% (7/107) incidence of low-grade intraepithelial neoplasia in colorectal juvenile polyps. Unlike in cases of CSM-related adenoma, CSM was not a tumorigenic marker in cases of colorectal juvenile polyps. Juvenile polyps may progress from low-grade intraepithelial neoplasia to high-grade intraepithelial neoplasia and then to carcinoma and should be treated cautiously when discovered, with regular follow-up as for colorectal adenomas.

## Data Availability

The datasets used and/or analysed during the current study are available from the corresponding author on reasonable request.

## Abbreviations

CSM: Chicken skin mucosa
JPS: Juvenile polyposis syndrome
CCS: Cronkhite–Canada syndrome
MLH1: MutL homologue 1
MSH2: MutS homologue 2
MSH6: MutS homologue 6
PMS2: Postmeiotic segregation increased 2
EMR: Endoscopic mucosal resection
COX-2: Cyclooxygenase-2
ESD: Endoscopic submucosal dissection
